# The various effect of social isolation on depression risk among old population in China during covid-19 pandemic: A population based survey

**DOI:** 10.1371/journal.pone.0325595

**Published:** 2025-06-06

**Authors:** Zhenjie Wang, Yongai Jin, Wanning Tian, Xun Zhou

**Affiliations:** 1 Institute of Population Research, Peking University, Beijing, People’s Republic of China,; 2 Center for Population and Development Studies, School of Population and Health, Renmin University of China, Beijing, People’s Republic of China,; 3 School of Publishing, Beijing Institute of Graphic Communication, Beijing, People’s Republic of China; The Hong Kong Polytechnic University, HONG KONG

## Abstract

**Background:**

The aim of the current study is to assess various effect of social isolation on depression risk among older adults during COVID-19 in China.

**Methods:**

Data was obtained from the China Longitudinal Ageing Social Survey (CLASS) conducted in 2020. A total of 9883 participants were included. Depression status was assessed by 9-item Center for Epidemiological Studies Depression Scale (CES-D). Social isolation was assessed by Lubben Social Network Scale-6 (LSNS-6). The odds ratios of depression risk according to LSNS-6 categories were obtained using a logistic regression model with adjustment for potential confounding variables.

**Results:**

The prevalence of depression was 31.2%, and the presence of social isolation was 37.9% during the COVID-19 among older population. A decrease in depression risk was observed with reduced isolation. The odds ratio for the lowest versus highest was 0.75 (95% confidence interval: 0.63, 0.89; *P*_trend _= 0.012). Friend support reduced depression risk more significantly than family support. The association between LSNS-6 friend subscale and depression risk was differentiated by LSNS-6 family subscale. In men, LSNS-6 friend subscale tended to be associated with depression risk inversely when their LSNS-6 family subscale was less than 6 (interaction *P* = 0.041). Similar associations and stratified modifications were observed among those who lived in rural areas (interaction *P* = 0.002), married (interaction *P* = 0.003), Han (interaction *P* = 0.01), lived with others (interaction *P* = 0.001), and so on.

**Conclusions:**

Most depression cases were found to be strongly associated with social isolation during the pandemic. Our findings have provided empirical evidence for researchers to understand the association between social isolation and depression, which could help them evaluate and manage depression promptly. Because of the cross-sectional design, we cannot establish causal relationships between depression and isolation.

## Introduction

Depressive disorder, defined by extended bouts of low mood or a loss of interest in activities once enjoyed, is a common mental health condition [1]. It is estimated that nearly one in eight people worldwide have a mental illness, and almost half of all individuals will experience mental illness at some point in their lives [2].

The global mental health landscape has been significantly altered by the COVID-19 pandemic [3]. Measures implemented to curb the virus spread have markedly affected individuals’ mental health [4,5]. While many have adjusted to the pandemic’s challenges [6], a significant portion of the population has continued to grapple with mental health issues. A 25% surge in global anxiety and depression prevalence was reported by the WHO in the first year of the COVID-19 pandemic [2]. This increase has particularly impacted older adults, as isolation, health worries, and fears of illness or death may contribute to a higher depression prevalence. During the COVID-19 pandemic, the depression prevalence among the elderly was reported as 26.4% in the China [7]. A 2020 online survey found a 30.8% prevalence among China’s elderly population [8].

Social isolation and loneliness represent the objective and subjective aspects of weak social relationships [9]. Social isolation, the actual lack or near lack of social relationships or connections, is a quantitative measurement of network size, diversity, and contact frequency [10]. Positive social relationships are health-protective, and ample evidence suggests that weak social relationships can lead to various adverse health outcomes [11–13], with depression being a primary concern. Numerous studies have respectively linked social isolation and loneliness to depressive symptoms [14–17].

Currently, there is limited epidemiologic evidence linking isolation with depression risk among Chinese older population during the COVID-19 pandemic. Therefore, we investigated the association between isolation and depression risk using data from the Chinese Longitudinal Ageing Social Survey (CLASS) in China.

## 2. Methods

### 2.1 Study population

In the current study, we used the data from the Chinese Longitudinal Ageing Social Survey (CLASS) 2020 collected by the National Survey Research Center at Renmin University of China. The CLASS was a large-scale, nationally representative survey, which covered 28 provincial regions in China. The survey was conducted face-to-face by trained interviewers. A stratified, multi-stage, probabilistic sampling method was used to select subjects in the CLASS. During the survey, 134 counties were first randomly selected as the primary sampling units, and then 462 communities were randomly selected from these primary sampling units. All households in each selected community were mapped and a random sample of 25 households was selected. Lastly, an elderly aged 60 or above from each randomly selected household was surveyed, which yielded a total of 11,398 older adults surveyed. In the present study, the sample is comprised of 9883 subjects aged 60 or above who had answered the questions on depressive symptoms and other independent variables of interest.

### 2.2 Depression assessment

Depression was assessed using an abbreviated nine-item Center for Epidemiological Studies Depression Scale (CES-D), which is reliable and valid for detecting non-psychotic mental disorders among Chinese older adults [18]. Among the nine items, three items assessed positive feelings (feeling happy, enjoying life, feeling pleasure), two items assessed negative emotions (feeling lonely, feeling upset), two items assessed somatic symptoms (having a poor appetite, having trouble sleeping), and two items assessed sense of marginalization (feeling useless, having nothing to do). Each item had a score of 0 (rarely or none of the time), 1 (some of the time), or 2 (most of the time), with the total score ranging from 0 to 18. By reversing the coding of the positive effect items, a higher score indicates a higher level of depressive symptoms. Using Kohout’s formula [19], standardized cut scores are determined by dividing the total possible score on a short CES-D scale by 60 (the total possible score on the full 20-item CES-D) and multiplying that number by 16 (the established cut score on the full 20-item CES-D). For current study, on a 9-item scale, the total possible score is 18 (9 items multiplied by 2, the highest response). That total score is divided by 60, which equals 0.3. Then, the 0.3 is multiplied by 16, resulting in a standardized cut score of 4.8 for the 9-item form of the CES-D. In this study, the internal Cronbach’s alpha for the nine items was 0.75.

### 2.3 Social isolation assessment

A detailed description of the individual’s social isolation was obtained via the following three indicators: 1) marital status, 2) living arrangement, and 3) social connectedness with relatives and friends measured by the Lubben Social Network Scale-6 (LSNS-6). The LSNS-6 measures the size, closeness and frequency of contacts of a participant’s social network with reference to the level of perceived support they receive from relatives (socially connected with relatives) and friends (socially connected with friends). The LSNS-6 provides quantitative information on kinship (extended family) and friendship ties and thus may be classed as “objective” measures [20]. Each item was scored from 0 to 5. The scores for each item were added up to produce a total score of LSNS-6 ranging from 0 to 30, with lower scores indicating isolation increase. The total score of the 6 items was divided into quartiles for the analysis of the relationship between social isolation indicators and loneliness. Those in the first quartile (score ranged from 0 to 12) were considered isolated, those in the second (score ranged from 13 to 16) were at high risk of isolation, those in the third (score ranged from 17 to 20) were at moderate risk of isolation, and those in the fourth (scored 21 and above) were at low risk of isolation [21,22]. The LSNS-6 and its two subscales have demonstrated high levels of reliability (Cronbach’s alpha = 0.80 ± 0.89), stable factor structures, and high correlations with criterion variables [20].

### 2.4 Independent variables of interest

We included the following socioeconomic characteristics in our study: age (60–64, 65–69, 70–74, 75–79, 80+), gender (male, female), residence (rural, urban), marital status (married, widowed/divorced/unmarried), education level (junior high school and above, primary school, never attended school), ethnicity (Han, Others), and living arrangements (living alone, living with others). Health variables were comprised of physical disability and chronic illness. Physical disability was assessed using the ten-item version of the activities of daily living (ADL) scale [23]. The ADL scale assesses a person’s capacity to clean, bathe, dress, go to the toilet, eat, transfer, climb stairs, and walk alone as well as their tendency for aconuresis and encopresis. We dichotomized the disability status into two groups (“no functional problems” = 0, “has at least one limitation” = 1). The participants were also asked whether they had one of the given health problems (covered 23 chronic diseases), including hypertension (yes/no), diabetes mellitus (yes/no), arthritis (yes/no), cerebrovascular disease (yes/no), liver disease, and so on. The number of comorbid chronic diseases was further categorized into “0”, “1” and “≥2”.

### 2.5 Statistical analysis

The difference between subjects with/without depression was tested by χ^2^ test with proportions. The logistic regression model was used to calculate the adjusted odds ratios (OR) and 95% confidence interval (CI) of depression (yes/no) with the covariates: age group, gender, residence, marital status, education level, ethnicity, physical disability, number of comorbid chronic diseases, and living arrangement. The linear trend of the association was assessed with ordinal scores 0–3 assigned to four categories of isolation and with ordinal scores 0–1 assigned to LSNS-6 Friend Subscale (< 6 and ≥ 6) in order, respectively.

Quartile categories of the isolation and LSNS-6 Friend Subscale (< 6 and ≥ 6) were used in the stratified analysis, because the number of the subjects was smaller within strata. Stratified analysis was conducted with respect to gender (male, female), residence (rural, urban), marital status (married, widowed/divorced/unmarried), ethnicity (Han, Others), living arrangements (living alone, living with others), physical disability (no functional problems, has at least one limitation), the number of comorbid chronic diseases (0, 1, ≥ 2), the combination of LSNS-6 family subscale (< 6 and ≥ 6) and interested variables (mentioned above). Interaction was evaluated by the Wald statistic for the interaction term, i.e., a product of an ordinal variable for the isolation score and a dichotomous variable for stratification. Statistical significance was declared with a two-sided *p*-value < 0.05. Statistical analyses were performed using SAS version 9.2 (SAS Institute Inc., Cary, NC, USA).

## Results

Selected characteristics of the subjects with/without depression are summarized in Table 1. In the current study, the prevalence rate of depression was 31.2%. The presence of social isolation was found in 3748 (37.9%), family isolation in 3584 (36.3%) and friend isolation in 4620 (46.7%) participants. Male subjects, urban residents, people living with others, and the Han nationality accounted for a majority of the Chinese elderly population. Compared with the subjects without depression, the depression cases were slightly older and had a higher proportion of female subjects and of physical disability, while a lower proportion of them are living with others.

**Table 1:** Subjects’ characteristics by their depression status according to the nine-item Center for Epidemiological Studies Depression Scale (CES-D) among elder population in China.

Variables	Without depression *n* (%)	With depression *n* (%)	*P*
Age groups			
60-64	987 (14.5)	296 (9.6)	< 0.001
65-69	2296 (33.8)	907 (29.4)	
70-74	1813 (26.7)	817 (26.5)	
75-79	919 (13.5)	508 (16.5)	
80+	784 (11.5)	556 (18.0)	
Gender			
Male	3503 (51.5)	1470 (47.7)	< 0.001
Female	3296 (48.5)	1614 (52.3)	
Residence			
Urban	3471 (51.1)	1681 (54.5)	0.001
Rural	3328 (48.9)	1403 (45.5)	
Marital status			
Married	5316 (78.2)	2160 (70.0)	< 0.001
Widowed/divorced/ Unmarried	1483 (21.8)	924 (30.0)	
Education level			
Never attended school	1657 (24.4)	1005 (32.6)	< 0.001
Primary school	2460 (36.2)	1161 (37.6)	
Junior High school and above	2682 (39.4)	918 (29.8)	
Ethnicity			
Han	6439 (94.7)	2870 (93.1)	0.001
Others	360 (5.3)	214 (6.9)	
Living arrangement			
Live with others	6173 (90.8)	2710 (87.9)	< 0.001
Live alone	626 (9.2)	374 (12.1)	
Physical disability			
No function problems	5675 (83.5)	2305 (74.7)	< 0.001
One and more functioning limitations	1124 (16.5)	779 (25.3)	
Number of comorbid chronic disease			
0	1634 (24.0)	539 (17.5)	< 0.001
1	1997 (29.4)	851 (27.6)	
≥2	3168 (46.6)	1694 (54.9)	
Lubben Social Network Scale-6 (LSNS-6), mean (SD)	14.3 (5.12)	13.7 (5.05)	< 0.001
LSNS-6 Family Subscale, mean (SD)	7.4 (2.63)	7.3 (2.66)	0.005
LSNS-6 Friend Subscale, mean (SD)	6.8 (3.01)	6.4 (2.92)	< 0.001

**Fig 1:**
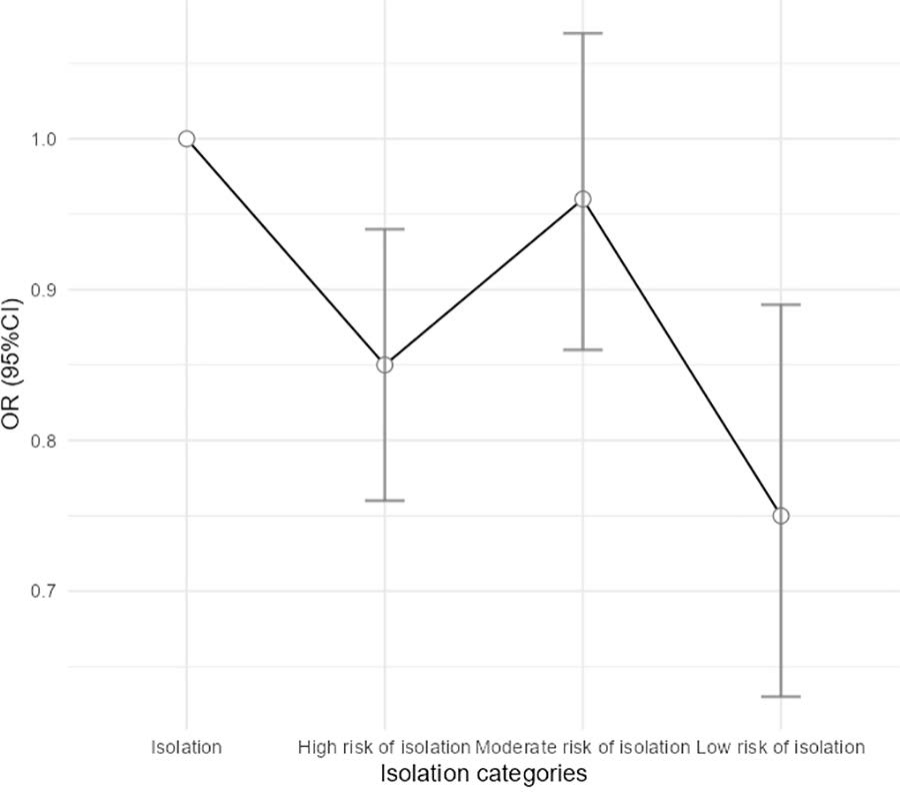
Odds ratio (95% confidence interval) of depression risk according to Lubben Social Network Scale-6 (LSNS-6) score rage^*.^ ^*^ Adjusted for age, residence, marital status, education level, ethnicity, living arrangement, physical disability, number of comorbid chronic disease.

The unadjusted OR and adjusted OR for different variables considered were presented in Table 2. The unadjusted OR (95% CI) for the low risk of isolation to the high risk of isolation were 1.00 (reference), 1.50 (1.26–1.77), 1.24 (1.04–1.48), and 1.41 (1.18–1.69), respectively (*P*_trend_ <0.001). The multivariate-adjusted OR (95% CI) for the low risk of isolation to the high risk of isolation were 1.00 (reference), 1.34 (1.13–1.59), 1.13 (0.95–1.35), and 1.28 (1.07–1.54), respectively (*P*_trend_ = 0.012). The multivariate-adjusted OR (95% CI) for the high risk of isolation to the low risk of isolation were presented in the Fig 1. LSNS-6 score range, age, ethnicity, marital status, education level, physical disability, and number comorbid chronic disease were significantly associated with depression risk.

**Table 2:** Odds ratio (95% confidence interval) of depression risk according to characteristics^*.^

	Unadjusted OR (95%CI)	Model 1	Model 2	Model 3
Lubben Social Network Scale-6 (LSNS-6) score rage				
Low risk of isolation (21-)	Reference	Reference	Reference	Reference
Moderate risk of isolation (17–20)	**1.50 (1.26-1.77)**	**1.46 (1.23-1.73)**	**1.40 (1.18-1.66)**	**1.34 (1.13-1.60)**
High risk of isolation (13–16)	**1.24 (1.04-1.48)**	**1.22 (1.02-1.45)**	**1.21 (1.01-1.44)**	1.14 (0.95-1.36)
Isolation (0–12)	**1.41 (1.18-1.69)**	**1.37 (1.14-1.63)**	**1.35 (1.13-1.62)**	**1.29 (1.07-1.54)**
Age groups				
60-64	Reference	Reference	Reference	Reference
65-69	**1.32 (1.13-1.53)**	**1.30 (1.14-1.54)**	**1.24 (1.07-1.45)**	**1.22 (1.05-1.42)**
70-74	**1.50 (1.29-1.75)**	**1.50 (1.28-1.75)**	**1.34 (1.14-1.57)**	**1.27 (1.08-1.48)**
75-79	**1.84 (1.56-2.18)**	**1.83 (1.54-2.17)**	**1.58 (1.33-1.88)**	**1.42 (1.19-1.70)**
80+	**2.37 (2.00-2.80)**	**2.35 (1.98-2.78)**	**1.89 (1.58-2.26)**	**1.61 (1.34-1.93)**
Gender				
Men	Reference	Reference	Reference	Reference
Women	**1.17 (1.07-1.27)**	**1.17 (1.07-1.27)**	1.08 (0.99-1.18)	1.06 (0.97-1.16)
Ethnicity				
Others	Reference	Reference	Reference	Reference
Han	**0.75 (0.63-0.89)**	**0.76 (0.34-0.91)**	**0.79 (0.66-0.95)**	**0.79 (0.66-0.95)**
Residence				
Urban	Reference		Reference	Reference
Rural	**0.87 (0.80-0.95)**		1.00 (0.91-1.10)	0.97 (0.88-1.07)
Marital status				
Widowed/divorced/ Unmarried	Reference		Reference	Reference
Married	**0.65 (0.59-0.72)**		**0.81 (0.72-0.91)**	**0.85 (0.75-0.96)**
Education level				
Never attended school	Reference		Reference	Reference
Primary school	**0.78 (0.70-0.86)**		**0.87 (0.78-0.96)**	0.90 (0.80-1.00)
Junior High school and above	**0.56 (0.51-0.63)**		**0.69 (0.61-0.78)**	**0.72 (0.64-0.82)**
Living arrangement^†^				
Live with others	Reference		Reference	Reference
Live alone	**1.36 (1.19-1.56)**		1.04 (0.89-1.22)	1.13 (0.96-1.33)
Physical disability^†^				
No function limitation	Reference			Reference
One and more function limitations	**1.71 (1.54-1.89)**			**1.36 (1.22-1.52)**
Number of comorbid chronic disease^†^				
0	Reference			Reference
1	**1.29 (1.14-1.47)**			**1.18 (1.04-1.34)**
≥2	**1.62 (1.45-1.82)**			**1.36 (1.21-1.54)**

Abbreviations: LSNS = Lubben Social Network Scale; CI = confidence interval; OR = odds ratio.

* Variables in Model 1: age group, gender, and ethnicity; Variables in Model 2: age group, gender, ethnicity, marital status, education level, and living arrangement; Variables in Model 3: age group, gender, ethnicity, marital status, education level, living arrangement, physical disability, and number of comorbid chronic diseases.

The associations between subjects’ characteristics and depression risk were presented in Table 3. Depression risk increased significantly among those who were living alone or with one and more limitations. The associations between isolation and depression risk significantly differed because of physical disability and the number of comorbid chronic diseases. Low risk of isolation tended to decrease depression risk among those with one and more functional limitations.

**Table 3:** Odds ratio (95% confidence interval) of depression risk according to Lubben Social Network Scale-6 (LSNS-6) score rage.

	Isolation	High risk of isolation	Moderate risk of isolation	Low risk of isolation	*P*_trend_	*P*_*interaction*_
	*n = *3748	*n = *2816	*n = *2478	*n = *841		
Lubben Social Network Scale-6 (LSNS-6) score rage	0-12	13-16	17-20	21-		
Gender^†^						
Men	Reference	**0.80 (0.69-0.94)**	1.00 (0.85-1.17)	**0.78 (0.61-0.98)**	0.173	0.532
Women	1.06 (0.92-1.21)	0.94 (0.81-1.09)	0.98 (0.84-1.14)	**0.75 (0.58-0.97)**	**0.024**	
Residence^†^						
Urban	Reference	**0.78 (0.67-0.91)**	0.86 (0.74-1.00)	**0.74 (0.59-0.93)**	0.586	0.136
Rural	0.87 (0.75-1.00)	**0.81 (0.69-0.94)**	0.94 (0.80-1.10)	**0.65 (0.49-0.85)**	**0.005**	
Marital status^†^						
Widowed/divorced/ Unmarried	Reference	0.90 (0.73-1.10)	0.86 (0.70-1.07)	0.81 (0.56-1.18)	0.109	0.691
Married	0.86 (0.72-1.02)	**0.71 (0.60-0.85)**	0.85 (0.71-1.02)	**0.63 (0.50-0.79)**	0.054	
Ethnicity^†^						
Others	Reference	**0.55 (0.35-0.86)**	**0.57 (0.38-0.87)**	0.57 (0.27-1.19)	**0.021**	**0.046**
Han	**0.58 (0.44-0.78)**	**0.51 (0.38-0.68)**	**0.58 (0.43-0.78)**	**0.44 (0.32-0.61)**	0.055	
Living arrangement^†^						
Live with others	Reference	**0.87 (0.78-0.98)**	1.01 (0.91-1.34)	**0.75 (0.62-0.89)**	0.074	0.052
Live alone	1.33 (1.08-1.64)	0.92 (0.68-1.23)	0.77 (0.55-1.08)	1.20 (0.64-2.62)	**0.005**	
Physical disability^†^						
No function limitation	Reference	**0.75 (0.67-0.85)**	**0.83 (0.73-0.94)**	0.67 (0.56-0.81)	**<0.001**	**<0.001**
One and more function limitations	0.92 (0.77-1.09)	1.19 (0.99-1.44)	**1.51 (1.24-1.85)**	1.05 (0.71-1.57)	**0.005**	
Number of comorbid chronic disease^†^						
0	Reference	0.90 (0.70-1.60)	0.92 (0.71-1.18)	**0.64 (0.46-0.89)**	**0.024**	**0.001**
1	**1.37 (1.13-1.67)**	0.90 (0.72-1.12)	0.95 (0.76-1.18)	0.74 (0.52-1.06)	**<0.001**	
≥2	1.19 (0.99-1.39)	1.15 (0.95-1.39)	**1.40 (1.16-1.71)**	1.20 (0.91-1.59)	0.210	

Abbreviations: LSNS = Lubben Social Network Scale; CI = confidence interval; OR = odds ratio.

* Adjusted for age, residence, marital status, education level, ethnicity, living arrangement, physical disability, number of comorbid chronic disease.

† Adjusted for age, residence, marital status, education level, ethnicity, living arrangement, physical disability, number of comorbid chronic disease, except for selected stratified variable.

**Fig 2:**
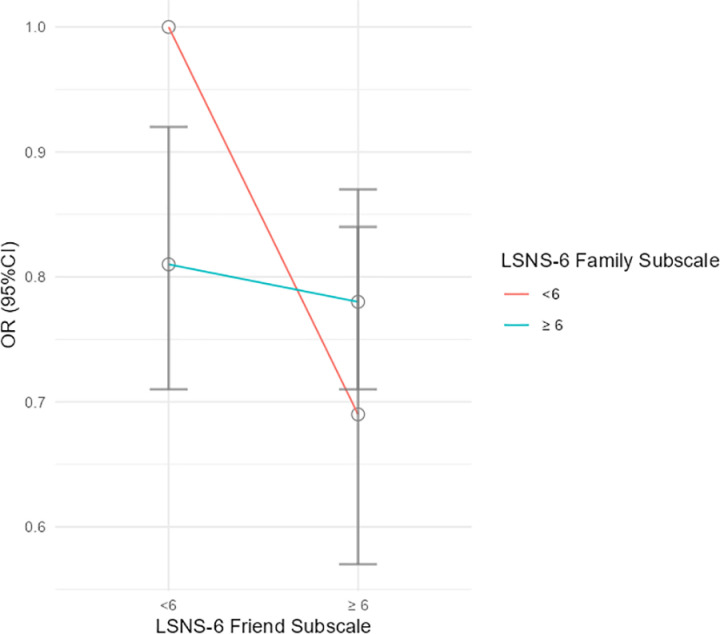
Odds ratio (95% confidence interval) of depression according to combined categories of LSNS-6 family subscale and LSNS-6 friend subscale^*^ ^.^^*^ Adjusted for age, residence, marital status, education level, ethnicity, living arrangement, physical disability, number of comorbid chronic disease.

The various effect of different combination of LSNS-6 family subscales and LSNS-6 friend subscales on depression risk were presented in Table 4. Friend support was found to be more effective than family support in reducing the risk of depression, with the reduction ranging from 3% to 31%. Overall, high LSNS-6 family subscale significantly decreased the depression risk by 19% among those with low LSNS-6 friend subscale. Moreover, the high LSNS-6 friend subscale significantly decreased the depression risk by 22% and 31% among different LSNS-6 family subscale, respectively (Fig 2). Similar associations and significantly stratified modifications were observed among men, those who lived in rural areas, married, Han, lived with others, without functional limitation, and had more than 2 comorbid chronic diseases.

**Table 4:** Odds ratio (95% confidence interval) of depression according to combined categories of LSNS-6 family subscale and LSNS-6 friend subscale.

	LSNS-6 Family Subscale	LSNS-6 Friend Subscale, < 6		LSNS-6 Friend Subscale, ≥ 6		*P*_trend_	*P*_interaction_
		n	OR (95%CI)	n	OR (95%CI)		
Gender^†^							
Men	< 6	1534	Reference	322	**0.69 (0.53-0.92)**	**0.015**	**0.041**
	≥6	813	**0.82 (0.68-0.99)**	2304	**0.80 (0.69-0.92)**	0.952	
Women	< 6	1417	Reference	311	**0.69 (0.53-0.91)**	**0.014**	0.055
	≥6	856	**0.80 (0.67-0.96)**	2326	**0.76 (0.66-0.88)**	0.84	
Residence^†^							
Urban	< 6	1644	Reference	313	0.78 (0.60-1.02)	0.092	0.392
	≥6	823	0.87 (0.73-1.04)	2372	**0.78 (0.68-0.90)**	0.297	
Rural	< 6	1307	Reference	320	**0.62 (0.46-0.83)**	**0.001**	**0.002**
	≥6	846	**0.73 (0.60-0.89)**	2258	**0.78 (0.67-0.91)**	0.214	
Marital status^†^							
Widowed/divorced/ Unmarried	< 6	843	Reference	146	0.70 (0.48-1.02)	0.089	0.575
	≥6	425	0.91 (0.71-1.16)	993	**0.72 (0.59-0.88)**	0.137	
Married	< 6	2108	Reference	487	**0.69 (0.55-0.87)**	**0.002**	**0.003**
	≥6	1244	**0.77 (0.66-0.90)**	3637	**0.81 (0.72-0.91)**	0.349	
Ethnicity^†^							
Others	< 6	144	Reference	48	0.63 (0.30-1.33)	0.403	0.553
	≥6	95	0.78 (0.45-1.35)	287	**0.65 (0.42-0.99)**	0.616	
Han	< 6	2807	Reference	585	**0.72 (0.58-0.88)**	**0.002**	**0.01**
	≥6	1574	**0.81 (0.71-0.93)**	4343	**0.80 (0.72-0.88)**	0.94	
Living arrangement^†^							
Live with others	< 6	2496	Reference	560	**0.66 (0.54-0.82)**	**<0.001**	**0.001**
	≥6	1547	**0.80 (0.70-0.93)**	4280	**0.81 (0.73-0.91)**	0.569	
Live alone	< 6	455	Reference	73	0.93 (0.55-1.57)	0.803	0.193
	≥6	122	0.90 (0.59-1.37)	350	**0.53 (0.39-0.73)**	**0.03**	
Physical disability^†^							
No function limitation	< 6	2430	Reference	516	**0.70 (0.57-0.87)**	**0.002**	**0.011**
	≥6	1221	**0.70 (0.60-0.82)**	3813	**0.69 (0.62-0.78)**	0.857	
One and more functioning limitations 1	< 6	521	Reference	117	0.69 (0.44-1.08)	0.108	0.157
	≥6	448	1.28 (0.98-1.67)	817	1.27 (1.01-1.60)	0.992	
Number of comorbid chronic disease^†^							
0	< 6	690	Reference	153	1.27 (0.86-1.87)	0.245	0.143
	≥6	302	0.84 (0.61-1.16)	1028	**0.74 (0.59-0.93)**	0.44	
1	< 6	926	Reference	168	0.80 (0.56-1.16)	0.241	0.345
	≥6	447	**0.63 (0.49-0.82)**	1307	**0.63 (0.52-0.76)**	0.909	
≥2	< 6	1335	Reference	312	**0.48 (0.36-0.65)**	**<0.001**	**<0.001**
	≥6	920	0.90 (0.75-1.07)	2295	0.89 (0.77-1.03)	0.854	

Abbreviations: LSNS = Lubben Social Network Scale; CI = confidence interval; OR = odds ratio.

*Adjusted for age, residence, marital status, education level, ethnicity, living arrangement, physical disability, number of comorbid chronic disease.

†Adjusted for age, residence, marital status, education level, ethnicity, living arrangement, physical disability, number of comorbid chronic disease, except for selected stratified variable.

## Discussions

This is one of the limited studies exploring various associations between depression and social isolation among older population during the covid-19 in China. Our findings show a strong association between a lower degree of isolation and a decreased depression risk, after accounting for most important confounders. Different associations between depression and isolation were stratified by physical disability and the number of comorbid chronic diseases. Family and friend support presented various effect on depression risk among older population in China during the pandemic.

Our prevalence rate aligns with previous studies conducted during the COVID-19 pandemic. In China, the prevalence of depression among the elderly ranged widely from 9.1% [24] to approximately 40% [25]. Another online survey in 2020 found a depression prevalence of 30.8% among elderly Chinese individuals [26]. A prior meta-analysis estimated the prevalence rate of depressive symptoms to be between 21% and 42% among the elderly population during the pandemic [27]. The prevalence of depression in China did not increased significantly during the first year of the COVID-19 pandemic as WHO reported that there was a 25% increase worldly [2]. The possible reasons were the timeliness and effectiveness of the Chinese government’s COVID-19 control measures. Mobility restrictions and social distancing effectively reduced public panic and protected high-risk groups including elderly individuals [28]. Previous studies have suggested that elderly individuals may exhibit higher levels of psychological resilience during the COVID-19 pandemic because of their life experiences [27,29].

The research findings indicate a high risk of depression amongst older adults experiencing loneliness. These results echo earlier studies that identified a correlation between feelings of loneliness and depression [30]. Past research also highlights that loneliness can potentially lead to the development of self-deprecating beliefs due to the absence of companionship, significant connections, and a sense of belonging, thereby reducing social interactions which may inhibit depression. Additionally, the biological effect of loneliness on stress response, such as impaired immune functionality and altered hypothalamic-pituitary-adrenal axis activity, contribute to the onset of depression, according to previous research [30]. It is advisable to encourage early detection and management of loneliness via social prescribing, facilitated social interaction, social skills development, psycho-education, and psychological therapies targeting negative thought patterns to prevent depression in older adults. Our research also indicates that poor health in older adults is linked to a high risk of loneliness due to weakened immune function, which heightens the risk of physical and psychological issues among this demographic [30]. Therefore, initiatives that enhance companionship and a sense of belonging, and foster quality and meaningful relationships among older adults can help mitigate the adverse effects of loneliness, promoting better health and improving life quality [31].

Support from family has consistently acted as a safeguard against depression in the elderly population. However, in contrast to earlier life stages, the protective influence of family support seems less significant among older individuals. Only about one third of studies exploring family support found a notable link. As individuals age, the role of family and relatives may transform, given that relatives also age (or may pass away), potentially diminishing their capacity to provide support. Throughout different life stages, it is observed that support from friends is most consistently linked to a decrease in depression among the elderly. The assistance from friends can supplement the support received from a spouse or family. It is plausible that as people grow older and spouses and family become less available due to ageing, sickness or death, the value of friendship and companionship escalates. Our research, which stratified samples based on factors like gender and living arrangements, supports this pattern of evidence. Fiori et al. also discovered a significant correlation between positive support from friends and lower levels of depressive symptoms in older adults (aged 60 + years) [32]. Similarly, Okun & Keith observed that support from friends and/or relatives (excluding spouses or children) was significantly tied to fewer depressive symptoms in older adults (60–92 years old), a trend not seen in younger adults [33].

## Limitation

Our study boasts several strengths, including a large sample size, a population-based design, and adjustments for an extensive range of socioeconomic factors. However, potential limitations should be noted, so as to be able to guide future research. The retrospective assessment of depression is a problem inherent to cross-sectional studies. Additionally, the selection of participants may have introduced a selection bias. Moreover, given the cross-sectional nature of our study, we cannot establish causal relationships between depression and isolation. Therefore, future longitudinal studies are necessary to examine the causal relationships between isolation and depression. Once longitudinal data is available, we can determine causality with the foundation built by this study. Additionally, the CLASS survey does not provide adequate information on lifestyle factors such as weight, height, smoking habit, alcohol consumption, etc., which have been suggested as potential risk factors for depression. Future studies might be able to take these factors into account.

## Conclusions

China has already had the world’s largest older population (≥60 years old), and China has often been held up as an example for other middle-income countries. Given the challenges posed by the rapid population ageing in China, there is a distinct need for enhancing depression recognition in primary healthcare settings for old population. Our study’s findings carry significant implications for community-based programs, policy formulation and future research. In this study, depression cases were evaluated using the Center for Epidemiologic Studies Depression Scale (CES-D). Our findings provide empirical evidence for researchers to consider social support or social isolation as potential conditions for depression. This perspective could aid in the timely evaluation and management of depression, especially during a pandemic.

## Supporting information

S1 Table Fig1 data. (DOCX)

S2 Table Fig 2 data. (DOCX)

## Abbreviations

CLASS: China Longitudinal Ageing Social Survey
CES-D: Center for Epidemiological Studies Depression Scale
