# Supplementary material for: The various effect of social isolation on depression risk among old population in China during covid-19 pandemic: A population based survey
Source: PLoS One. 2025 Jun 6;20(6):e0325595. doi: 10.1371/journal.pone.0325595 (PMC12143535; doi:10.1371/journal.pone.0325595)
Supplement: S1 Table — (DOCX) [file pone.0325595.s001.docx]

**S1 Table. Fig1 data**

|  | Isolation | High risk of isolation | Moderate risk of isolation | Low risk of isolation | *P*_trend_ | *P_interaction_* |
| --- | --- | --- | --- | --- | --- | --- |
|  | *n=*3748 | *n=*2816 | *n=*2478 | *n=*841 |  |  |
| Lubben Social Network Scale-6 (LSNS-6) score rage | 0-12 | 13-16 | 17-20 | 21- |  |  |
| Overall**^*^** | **Reference** | **0.85 (0.76-0.94)** | 0.96 (0.86-1.07) | **0.75 (0.63-0.89)** | **0.012** |  |
