# Supplementary material for: The various effect of social isolation on depression risk among old population in China during covid-19 pandemic: A population based survey
Source: PLoS One. 2025 Jun 6;20(6):e0325595. doi: 10.1371/journal.pone.0325595 (PMC12143535; doi:10.1371/journal.pone.0325595)
Supplement: S2 Table — (DOCX) [file pone.0325595.s002.docx]

**S2 Table. Fig2 data**

|  | LSNS-6 Family Subscale | LSNS-6 Friend Subscale, < 6 | | LSNS-6 Friend Subscale, ≥6 | | *P*_trend_ | *P*_interaction_ |
| --- | --- | --- | --- | --- | --- | --- | --- |
|  |  | n | OR (95%CI) | n | OR (95%CI) |  |  |
| **Overall**^*^ | < 6 | 2951 | Reference | 633 | 0.69 (0.57-0.84) | < 0.001 | 0.005 |
|  | ≥ 6 | 1669 | 0.81 (0.71-0.92) | 4630 | 0.78 (0.71-0.87) | 0.92 |  |
